# Enhanced Fire Retardancy of Epoxy Resins upon Addition of Boron Nitride Nanoparticles Using Boron Polyol Complex

**DOI:** 10.3390/ma18174101

**Published:** 2025-09-01

**Authors:** Lalson D. Mathews, Srikanth Mateti, Jyotishkumar Parameswaranpillai, Nishar Hameed, Nisa V. Salim

**Affiliations:** 1 School of Engineering, Swinburne University of Technology, Hawthorn, VIC 3122, Australia; ldanielmathews@swin.edu.au (L.D.M.); nisharhameed@swin.edu.au (N.H.); 2 Institute for Frontier Materials, Deakin University, Waurn Ponds, VIC 3216, Australia; s.mateti@deakin.edu.au; 3 AU-Sophisticated Testing and Instrumentation Center, Alliance University, Chandapura-Anekal Main Road, Anekal, Bengaluru 562106, Karnataka, India; jyotishkumar.p@alliance.edu.in

**Keywords:** boron nitride, epoxy resin, fire retardant, ionic liquid, thermal management

## Abstract

Fire retardancy and thermal management improvements in epoxy resins can critically impact their use in electronics for IoT and 5G devices. This study proposes a facile method to improve the fire retardancy and thermal properties of epoxy resins (EPs) by incorporating boron nitride nanoparticles (BNNPs) with boron polyol complex (BPC) to form an ionanofluid and explores the synergistic effect of polyelectrolytes with BN. The modified multifunctional additive BPC–BNNPs were then used for the functional modification of epoxy resin. Our detailed tests and analyses on these materials confirm that by adding 0.2 wt% of BNNPs in the EP–BPC–BN complex achieved a V-0 rating in the UL-94 vertical burning test. The resultant composite demonstrated that the modification of BN with the polyol complex imparted a low smoke and char formation in the modified epoxy composites. The current study shows that EP–BPC–BN complex has great potential as a thermal interface material for the thermal management of electronics or similar applications. The presented EP–BPC–BN composite can also be utilised as a fire-retardant coating, adhesive, and binding agent in the aerospace, transportation, and building industries.

## 1. Introduction

Epoxy resin is used widely in diverse industrial applications, ranging from building, construction, and manufacturing to electronics, defence, and space. Owing to their chemical resistance, adhesiveness, and excellent mechanical properties [1,2,3,4], epoxy resins are used as coatings, adhesives, paints, and high-performance materials. Unfortunately, the poor performance of epoxy resin in the event of fire restricts its all-encompassing applications. Epoxy resin is inherently flammable since it is a petroleum derivative, and it emits enormous amount of heat, smoke, and hazardous byproducts during combustion resulting in casualties and property losses [5]. Many strategies such as intrinsic chemical modification, blending with compounds, and incorporation of 2D or 3D nanomaterials [6] have been developed to address this issue. Epoxies blended with phosphorous, silicon, boron, or lignin compounds demonstrate appreciable fire-retardant properties [7,8,9]. Fire-retardant epoxy nano-composites based on graphene [10,11], carbon nanotubes [12,13], MXenes [14,15], benzoxazines [16], and clays [17] can improve the epoxy resin’s reaction to fire properties. Recently a bio-based flame-retardant additive synthesised from resveratrol was successfully utilised to impart fire-retardancy to epoxy resins [18]. Alternatively, the combination of several elements that provide a cooperative fire-retardant effect has been identified as a suitable method to improve the fire-retardant characteristics of epoxy resins. Recently, a phosphazene-based fire-retardant epoxy resin was synthesized by Terekhov et al. who identified that the synergetic effect of elements phosphorus and nitrogen enhanced one another’s effect in the combustion process of cured epoxy resins [19]. Similarly, halogenated epoxy-phosphazene oligomers in epoxy resin that contains elements chlorine or bromine and epoxy-phosphazene non-combustible oligomers can be used as a flame-retardant additive [20]. However, immiscibility, phase separation, difficulty in processing, deteriorated mechanical properties, environmental hazards, and limitations to scale up are the major associated challenges.

Because of the high rate of performance improvement, nanofillers are increasingly used as fire-retardant additives in epoxy matrix. The nanoparticles in the epoxy matrix enhance non-flammability characteristics such as increased catalytic carbonisation (char yield) [21], high thermal conductivity [22], delayed ignition time [23], and reduced heat release rate during combustion [24]. Similarly, micro fillers such as glass fibres and geopolymers enhance fire retardancy and mechanical properties of the epoxy resins by improved char formation and reduced emission of flammable hazardous gases [25]. Boron Nitride nanoparticles (BNNPs) are one of the advanced boron-based nano material fillers that are electrically nonconductive and are excellent additives to improve the flame retardancy of epoxy resins. In our previous research, we have successfully synthesised a boron-based fire-retardant epoxy blend with the use of boron polyol complex (BPC). The research findings confirmed that BPC forms hydrogen bonding with epoxy polymer chains which acts as a bridge for thermal conductivity. The increased flow of lattice vibrations—phonon transport—through the polymer chain via hydrogen bonding resulted in early thermal degradation of epoxy surface to form a stable layer of char. The carbonaceous char surface reduced the emission of combustible gases and protected the underlying epoxy layer from combustion to impart better fire retardancy [26]. In this research, we used traditional epoxy derived from bisphenol A and epichlorohydrin over bio-based epoxy resins due to its wide popularity in the industry for various applications. Additionally, we utilised BNNPs to develop an epoxy composite material, contrasting with the intrinsically modified epoxy blends reported in our previous research. We used BPC as a dispersion medium to introduce BNNPs to the epoxy matrix. The fire-retardant properties have further improved with less graphitisation and a high glass transition temperature with the addition of BNNPs along with BPC. The synergistic effect of BNNPs and BPC complex improved the overall thermal conductivity thorough H-bonding where filler contact was missing with lower loading. Since boron- and nitrogen-containing additives are flame retardants, a synergistic effect of BN flame retardant along with BPC as dispersion agent and H-bonding agent contributed to the excellent flame-retardant properties. We intend to modify bio-based epoxy resins with similar constituent materials in future research.

Generally, high loading of thermally conductive fillers is required for increased thermal conductivity [27] to achieve superior flame retardancy because, according to thermally conductive pathway theory (thermal conduction path theory) [28], the phonon transport path is connected through the contact of fillers which requires higher loading. However, higher loading is associated with poor dispersion and restricts formation of a continuous contact of the fillers [29]. The aggregated filler acts as a heat reservoir and restricts the heat diffusion [30]. Simple direct blending of BNNPs and epoxy monomer by sonication and vortex mixing results in BNNP aggregation due to the large surface area and strong van der Waals attraction [31]. Ali et al. identified that ultrasonic mixing did not disperse BNNPs in the polymer matrix completely and phase separation was observed [32]. He et al. reported that functionalisation of filler surface with covalently bonded functional groups to improve overall dispersion also results in structural defects of the filler [31]. To address these problems, we blended BNNPs with BPC to form a BPC–BN emulsion and introduced the BPC–BN to the epoxy matrix resulting in an extremely low concentration of BNNPs imparting excellent fire retardancy to the EP without aggregation.

Because of its exfoliation capacity, BPC can be used as a promising medium for the dispersion of BNNPs to the epoxy matrix. Such an idea has never been reported earlier. With respect to the unlimited applications of fire-retardant epoxy resins, this experiment focused on the development of an environmentally safe fire-retardant additive and investigated the molecular structure, reaction to fire, and thermal properties of the resultant fire-retardant epoxy composite.

## 2. Materials and Methods

### 2.1. Materials

The boron nitride (BN) nanoparticles used in this study were synthesized via high-energy ball milling of crystalline BN powder. A total of 4 g of BN was placed in the milling chamber along with four stainless steel balls of equal size. The milling was conducted under an inert argon atmosphere maintained at a pressure of 300 kPa to prevent oxidation or contamination. The milling speed was set at 160 rpm, and the process was carried out continuously for 20 h. As a result of the prolonged mechanical stress, the crystalline structure of the starting BN material collapsed, leading to the formation of BN nanoparticles. The final product consisted of non-functionalized particles, with no intentional surface charge or chemical groups, and an average size below 100 nm. Morphologically, the nanoparticles appeared near spherical to slightly irregular, consistent with materials produced through high-energy milling techniques.

Boric acid (100%), glycerol (100%), diglycidyl ether of bisphenol-A (DGEBA), and 4,4-diaminodiphenylmethane (DDM) were obtained from Sigma-Aldrich Pty. Ltd., Castle Hill, Australia. The epoxy equivalent weight of DGEBA was 172–176.

### 2.2. Synthesis of BPC Complex and Preparation of BNNP–BPC Colloid

**Figure 1:**
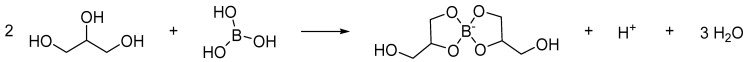
Synthesis of a boron chelate complex from boric acid and glycerol [26]. (Creative Commons CC BY license).

A 25% solution of boric acid in glycerol was prepared by mixing 17.5 g boric acid in 52.5 g glycerol at 40 °C under constant stirring until boric acid was completely dissolved. The mixture was denoted as boron polyol complex (BPC) and the synthesis was performed according to our previous research [26]. Refer to this work for the detailed characterisation of BPC complex and its blend with epoxy resin. Figure 1 represents the chemical equation for the synthesis of BPC complex from boric acid and glycerol. The mixture was placed in a water bath on a magnetic hotplate stirrer. The water bath was maintained and monitored at 40 °C. The solution was mixed for 2 h with a magnetic stirring bead. Various weights of BNNPs were added to 2 g BPC complex and sonicated for 8 h at 40 °C. The weight fractions of the components are listed in Table 1.

### 2.3. Preparation of EP–BPC–BNNP Blend and Composite

**Figure 2:**
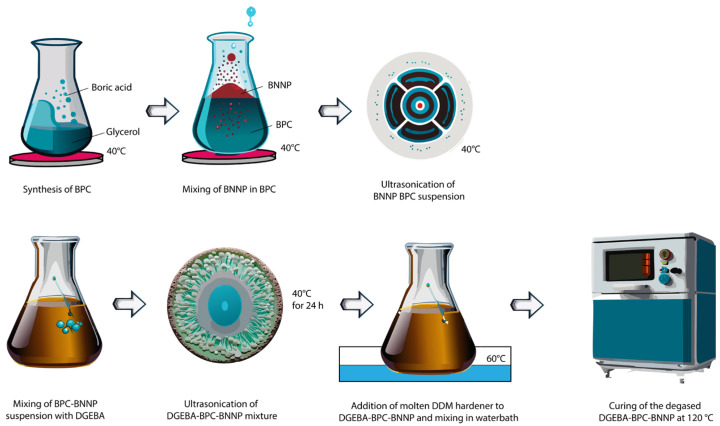
Schematic diagram of the synthesis of fire-retardant epoxy nanocomposite.

To synthesise the epoxy composite, BPC–BNNP suspension was added to 10 g DGEBA epoxy resin in centrifuge tubes and mixed thoroughly for 2 min with a vortex mixer. The solution was ultrasonicated in the bath for 8 hrs with the water temperature maintained at 40 °C for complete dispersion of the BNNP nanoparticles by inserting the centrifuge tubes in a rack and immersing the rack to the bath. The resultant mixture was treated with 3 g molten DDM curing agent [33] and vortex mixed for 2 min. The mixture was transferred immediately to a hot water bath for 2 min at 60 °C to maintain viscosity and vortex mixed for another 2 min. The mixture was then degassed and poured into a silicon mould and was cured in an oven at 120 °C for 24 h followed by post curing at 180 °C for 1 h. The mixing of the nanoparticles was controlled and monitored at various stages of preparation. A complete dispersion of BNNPs in BPC to form a nanofluid, followed by mixing the nanofluid with epoxy resin, was ensured by prolonged ultrasonication followed by visual observation. The ratio of BNNPs was estimated to be the maximum at 0.80 wt% since any further addition of BNNPs resulted in agglomeration indicating incomplete dispersion. Hence, those compositions were discarded from further characterization. The neat EP was denoted as EP-BPC-BN-0 and the blend of EP and BPC was denoted as EP-BPC-BN-1. All other compositions contained various wt% of BNNPs. Refer to Table 1 for details of the mixing ratio and Figure 2 is a schematic diagram of the process of synthesis.

### 2.4. Characterisations

Phase behaviour of the blend was investigated with a Netzsch Proteus 70 Differential Scanning Calorimeter (DSC) (Selb, Germany). The cured polymer samples were placed in an aluminium crucible and exposed to the first cycle of heating up to 100 °C with temperature ramp rate of 20 °C/min and held for 5 min at 100 °C. Then, the samples were cooled down to −30 °C and then reheated to 250 °C at 20 °C/min during the second cycle of heating. The glass transition temperature (*T*_g_) was measured as the temperature at the midpoint of the endothermic peak during the second cycle of heating.

The thermal diffusivity (α) of the specimens was measured with a laser flash thermal analyser (LFA) with sample size of 12.5 mm diameter and approximately 2.3 mm thickness. The specimens were spray coated with graphite powder on both sides before analysis.

The KBr method was used to determine the FTIR characteristics of the blend on a Bruker FTIR spectrometer (Ettlingen, Germany). The cured solid samples were placed on the clean surface of the sample holder. The spectra were recorded in the standard wavenumbers range of 400 and 4000 cm^−1^ at an average of 32 scans.

The specimens were tested for vertical flammability in general accordance with the UL-94 standard under laboratory conditions at a wind speed of 0.2–0.4 m/s and an ambient temperature of 23 °C.

Cone calorimetry testing was performed on plywood samples coated with neat EP and FREP in general accordance with the relevant [34]. Carbon fibre sheets fixed to the plywood surface with neat EP and FREP were also tested. Only one specimen was tested from each group.

The Raman spectra of the char layer and unburned blend were captured by a Renishaw inVia Raman instrument (Gloucestershire, UK). A 514 nm laser wavelength was selected to analyse graphitization. The morphology and the dispersion of the BNNPs in the epoxy were investigated using a Carl Zeiss Supra 55 VP Scanning Electron Microscope (SEM) instrument (Jena, Germany).

## 3. Results and Discussion

### 3.1. Phase Behaviour Studies of Neat Epoxy, Fire-Retardant Blend, and Composite

**Figure 3:**
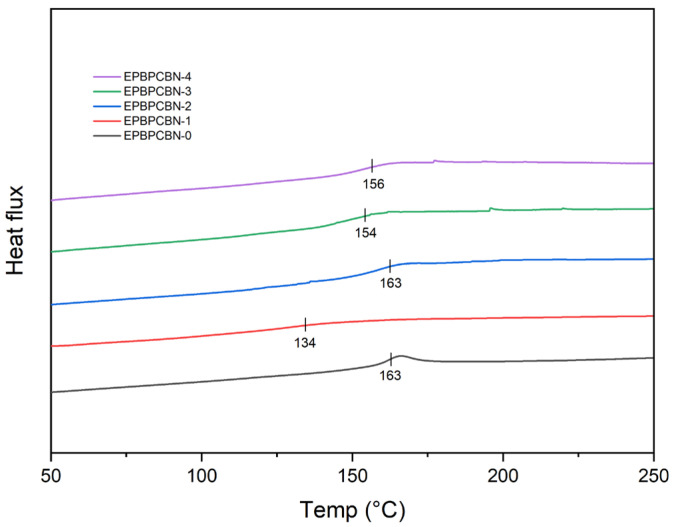
DSC diagram of neat EP, EP–BPC blend, and EP–BPC–BN composites. The x-axis represents the heating mode during the second cycle. The endothermic reaction near glass transition temperature is shown as peaks.

Figure 3 depicts the thermograms of neat EP, EP–BPC blend, and EP–BPC–BN composites. The glass transition temperature *T*_g_ of the neat EP was 163 °C. When the EP was blended with BPC, the *T*_g_ decreased to 134 °C as was proven in our previous research [26]. This is due to the increased free volume of DGEBA with the addition of BPC [35,36]. The decrease in *T_g_* value is also attributed to the lower degree of solidification [37], hydrogen bonding, steric hindrance, and the plasticisation effect of the polymer. When 0.2 wt% of BNNP was added to the blend, the *T_g_* of the EP–BPC blend increased to the original value of neat EP. This is attributed to the improved matrix–filler interface due to the reduced free volume. The uniform dispersion of BPC-assisted BNNPs in the EP matrix exhibits a strong confinement effect on the mobility of polymeric networks. It has been observed that there is a non-uniform and non-linear change in *T_g_* with the introduction of the same filler at increasing weight percentages. It appears that selective adsorption of epoxy resin components by the BNNPs and BPC leaves excess of hardener content and forms a difference in resin–glass interaction. An OH–BNNP interaction and OH–BPC interaction was observed in the FTIR analysis of the composite confirming the functional group interaction with the filler. The selective adsorption and participation of functional groups in adsorption interactions resulted in a decrease in *T_g_* [38]. Excessive addition of the filler may also result in poor interfacial interaction resulting in lower *T_g_* [39]. Moreover, the composite consists of BPC in addition to BNNPs in the epoxy matrix which tends to decrease the *T_g_* as confirmed in the EP-BPC-BN-1 specimen. Any further addition of more than 0.2 wt% of BNNPs did not support an increase in *T_g._*

### 3.2. Heat Propagation Studies of Neat Epoxy, Fire-Retardant Blend, and Composite

**Figure 4:**
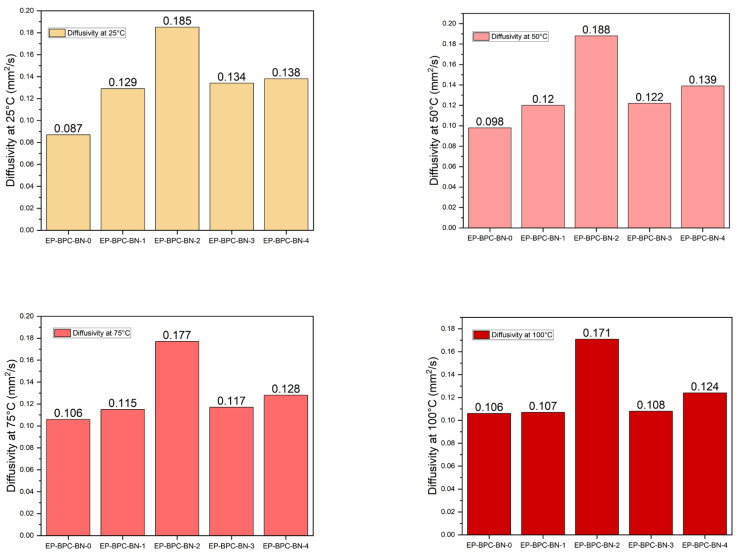
Thermal diffusivity of the specimens at 25 °C, 50 °C, 75 °C, and 100 °C.

**Figure 5:**
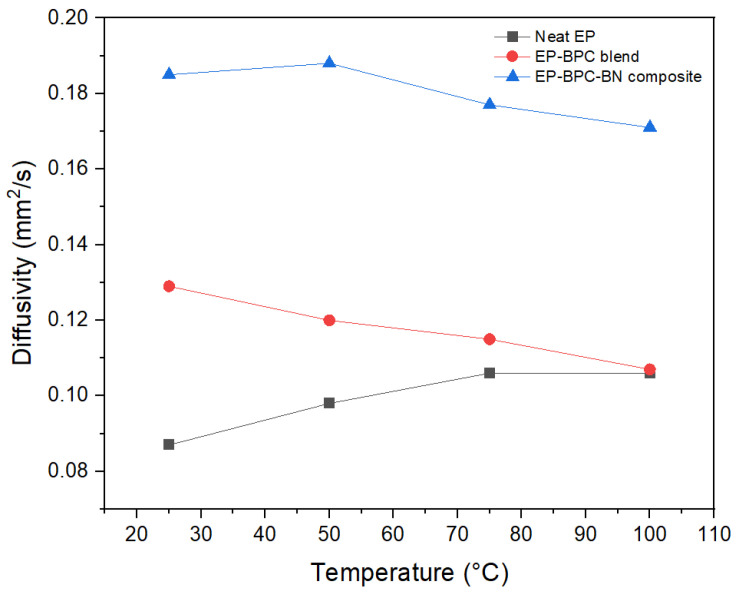
Thermal diffusivity changes of neat EP, EP–BPC blend, and EP–BPC–BN composite at 25 °C, 50 °C, 75 °C, and 100 °C.

The thermal diffusivity of the specimen perpendicular to the sample planar direction was measured by the laser flash technique. High thermal diffusivity indicates the ability of the composite to transmit heat rapidly. LFA results demonstrate that thermal diffusivity of the EP–BPC blend (0.13 mm^2^/s) was higher than neat EP (0.09 mm^2^/s). Epoxy resin shows low thermal diffusivity due to its amorphous nature. The thermal diffusivity of the blend was further increased to 0.19 mm^2^/s with the addition of 0.2 wt% of BNNPs. A similar pattern of linear increase in thermal diffusivity was observed when the experiment was carried out at 50 °C, 75 °C, and 100 °C (Figure 4). The enhanced thermal diffusivity is attributed to the increased thermal conduction pathways. A continuous passage of phonons through the BNNP nanoparticles in the epoxy matrix had resulted from the interaction between the epoxy matrix and BNNP fillers. Our study indicated the addition of BN into epoxies led a significant improvement in the thermal diffusivity compared to any other fillers such as carbon nanotubes [40,41]. This is attributed to the improved epoxy matrix–particle interface as a consequence of the exfoliation of BNNPs in the BPC matrix [42]. The change in thermal diffusivity of the neat EP, EP–BPC blend, and EP–BPC–BN composite is shown in Figure 5.

### 3.3. Vertical Burning Test

The flame-retardant properties of the neat EP and EP–BPC–BN composites were investigated using UL-94 vertical burning tests. The specimens were clamped in vertical position and a blue flame was applied for 10 s at the bottom free end. The test results are listed in Table 2.

**Figure 6:**
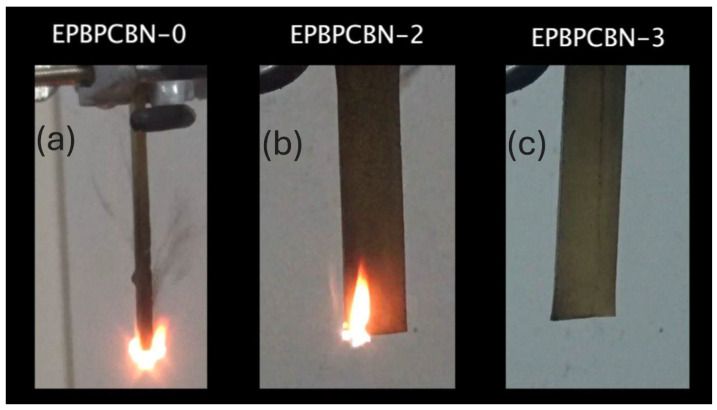
Images of (**a**) neat EP, (**b**) 0.2 wt% BNNP composite, and (**c**) 0.4 wt% BNNP composite, after 10 s of continuous exposure to flame.

**Figure 7:**
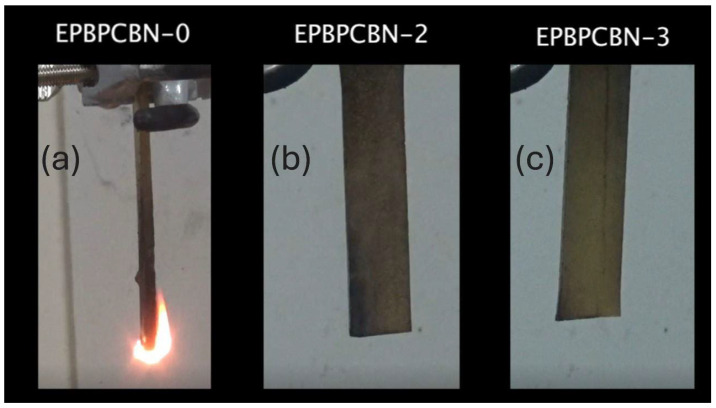
Images of (**a**) neat EP, (**b**) 0.2 wt% BNNP composite, and (**c**) 0.4 wt% BNNP composite, 2 s after 10 s of continuous exposure to flame.

**Figure 8:**
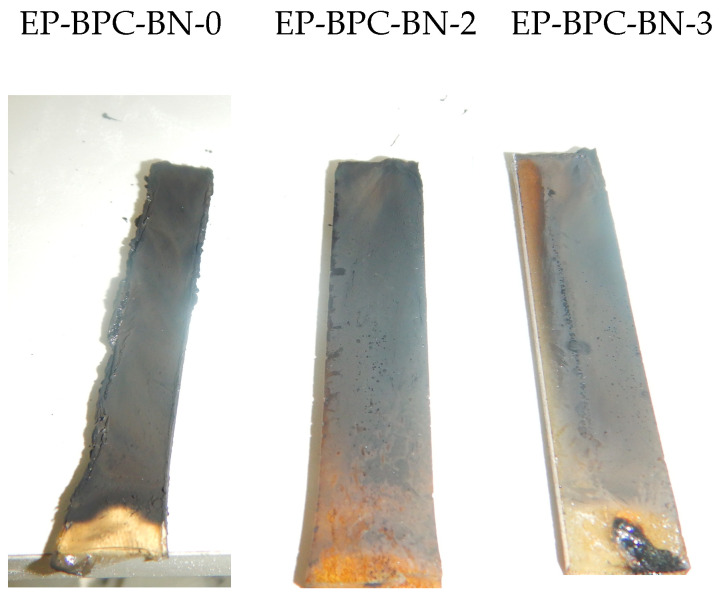
Images of EP and EP–BPC–BN composites after multiple ignitions in vertical burning test.

The flame had propagated through the complete height of neat EP and was covered with soft, unstable char. Neat EP failed to achieve a rating from the test. However, the EP–BPC blend obtained V-1 rating. Adding 0.2 wt% of BNNPs to the EP–BPC blend achieved V-0 rating and the flame extinguished in 2 s. With the further increase in BNNPs to 0.4 wt %, the composite maintained V-0 rating with immediate extinguishment of flame (Figure 6). Both composites did not have a significant amount of char on the surface and did not emit large amounts of smoke compared to the neat EP. The ignition of EP-BPC-BN-2 and EP-BPC-BN-3 composites was repeated to investigate their flammability, and it was observed that the composites self-extinguished without significant burning. Figure 7 shows images of the specimens 2 s after 10 s exposure to flame and Figure 8 shows images of the specimen after multiple events of ignition. The BNNP composites burned only at the bottom edge and the flame did not spread upwards. Figure 8 shows images of the specimens after multiple attempts of the vertical burning test.

### 3.4. Chemical Structural Analysis of the Burned Composite

**Figure 9:**
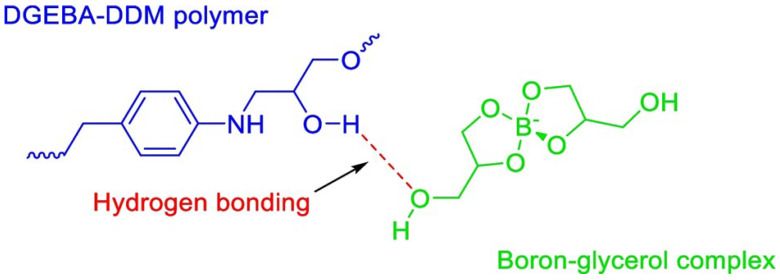
Formation of hydrogen bonding between the epoxy chain and BPC complex [26]. (Creative Commons CC BY license).

The BPC complex was initially characterized followed by the EP–BPC blend in our previous research [26]. It was found that the BPC complex forms a hydrogen bond with the epoxy chain as illustrated in Figure 9.

**Figure 10:**
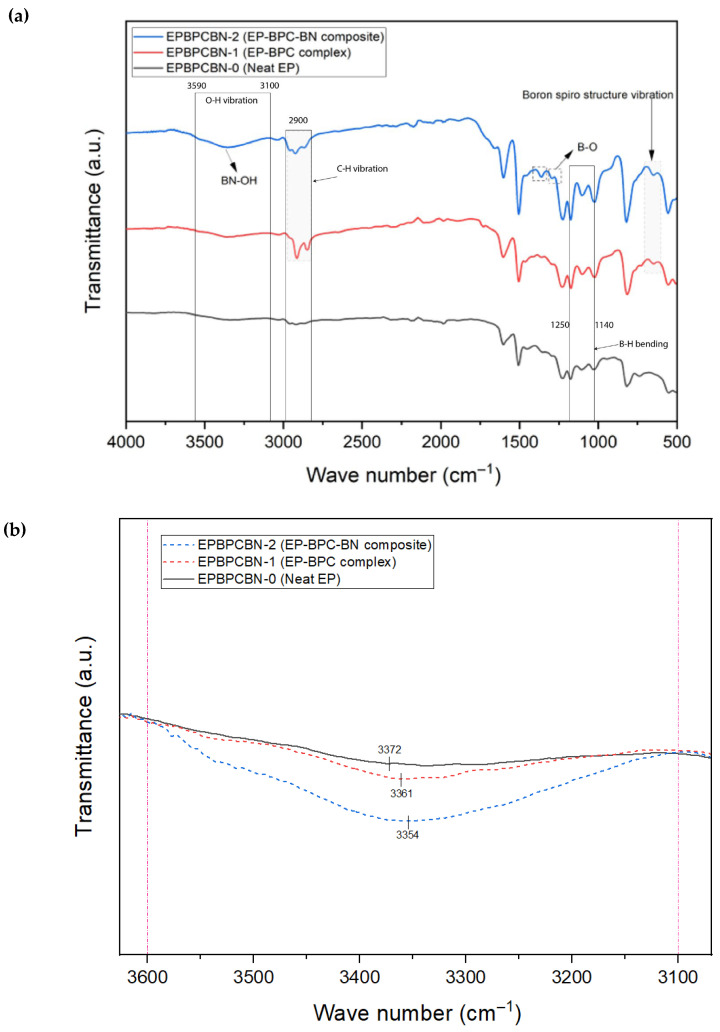
(**a**) FTIR spectra of the EP, EP–BPC complex, and EP-BPC-BN composite. (**b**) Region between 3100–3590 cm^−1^ illustrating peak shift towards lower wavelength representing hydrogen bonding and increased intensity of the peak with the addition of BPC and BNNPs representing higher concentration of O-H.

Figure 10a,b represents the FTIR spectra of the neat EP, EP–BPC complex, and EP–BPC–BNNP composite post burning. As shown in the figure, EP-BPC-BN-2 has two distinct absorption peaks around 1370 and 811 cm^−1^. The peak near 1370 cm^−1^ corresponds to the B-N tensile vibration within the sp^2^ bonding plane while the strong absorption peak at 811 cm^−1^ is attributed to the out-of-plane B-*N*-B bending vibrations. This is consistent with previously reported spectra [43]. This indicates the presence of non-combustible BNNPs in the epoxy matrix. In addition, the peak at 1290 cm^−1^ belongs to the B-O deformation vibration originating from the hydroxyl group of OH-BNNPs [44,45]. The absorption peaks observed in the range of 3100–3590 cm^−1^ correspond to O-H tensile vibrations. The peak at 3372 represents the self-associated hydroxyl groups in neat DGEBA, which shifted towards the lower region with the addition of BPC and BPC–BNNP nanofluid, confirming hydrogen bonding [46,47]. BPC complex being an ionic liquid when mixed with BN nanoparticles must have produced an ionanofluid which resulted in the formation of chemical bonding with the epoxy polymer matrix. The peak representing B-O-H bond was strong and broad in the EP–BPC–BN composite. This is attributed to the strength of BN-OH vibrations resulting from the formation of hydrogen bonds owing to the surface composition [45]. The peak shift and increase in intensity are represented in Figure 10b. Peaks in the range of 3100–3590 cm^−1^ and the peak at 1290 cm^−1^ indicate the functionalisation of BNNPs with the hydroxyl group and the increased hydrogen bonding between the hydroxyl groups of cured EP and BPC. The unique boron spiro structure vibration at 658 cm^−1^ was observed in both EP–BPC complex and EP–BPC–BN confirming the incorporation of BPC complex in the EP composite [48]. This was reconfirmed by the presence of C-H stretching vibrations at 2931 and 2850 cm^−1^ in both EP–BPC complex and EP–BPC–BN composite. Table 3 compares some of the specific peaks of burned EP, EP–BPC complex, and EP-BPC-BN composites.

### 3.5. Char Residue Analysis

**Figure 11:**
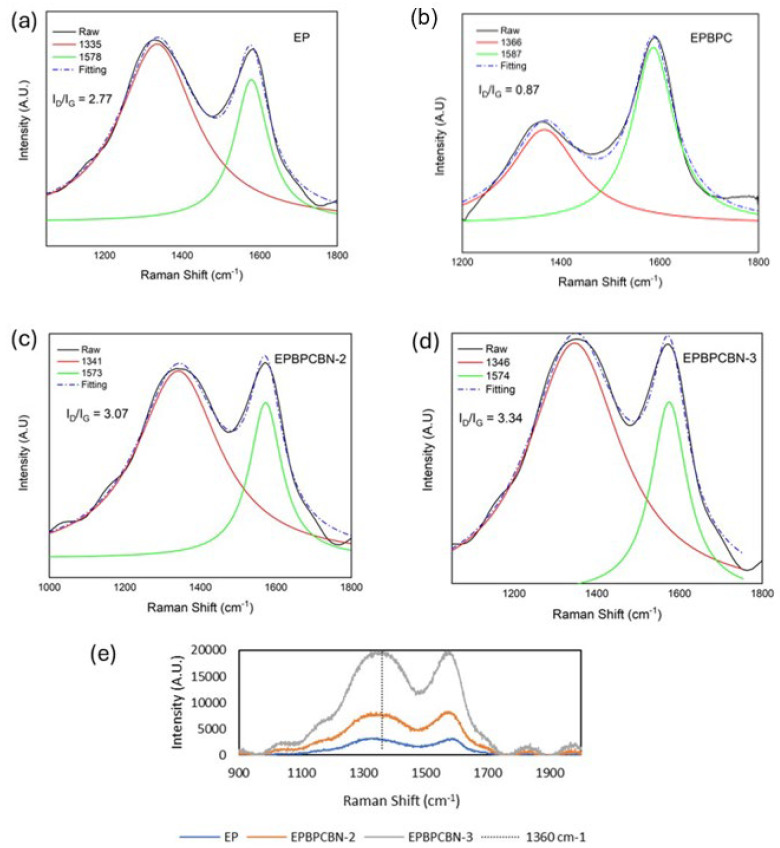
Raman spectra of (**a**) neat EP, (**b**) EP–BPC blend, and (**c**,**d**) EP–BPC–BN composites. (**e**) Comparison of BN characteristic peaks of the neat EP, EP-BPC-BN-2, and EP-BPC-BN-3.

Raman spectra of the burned surfaces of EP, EP–BPC blend and EP–BPC–BN composites were captured after the vertical burning test. There are two strong absorption peaks in the Raman spectra in the range of 1335–1346 cm^−1^ and 1573–1587 cm^−1^. These bands correspond to the peaks of graphite D and G bands. The I_D_/I_G_ value represents the degree of graphitisation of the carbon layer [49]. A lower I_D_/I_G_ value represents a higher degree of graphitisation. Figure 11 shows that the I_D_/I_G_ value of neat EP is 2.77 whereas the EP–BPC blend has a significantly lower value of 0.87 representing a high degree of graphitisation. The EP–BPC–BN composite has higher I_D_/I_G_ values compared to the neat EP due to a lower graphitisation degree. This is obvious in the post vertical burning test photographs in Figure 8. The lower char residue is due to the non-combustible BN nanoparticles. Figure 11e exhibits the characteristic peak of BN at ~1360 cm^−1^ due to the E_2g_ phonon mode [50] which confirms the presence of BNNPs in the char. A proportional increase in the intensities relative to BN concentration is obvious in the graph. A downward shift of the peak position form 1370 cm^−1^ to ~1360 cm^−1^ is attributed to the strain-induced BN stretching [50]. Unlike conventional fire-retardant additives, the BN–BPC nanocomposite product imparts excellent fire retardancy with less char formation.

### 3.6. Thermal Properties Analysis

**Figure 12:**
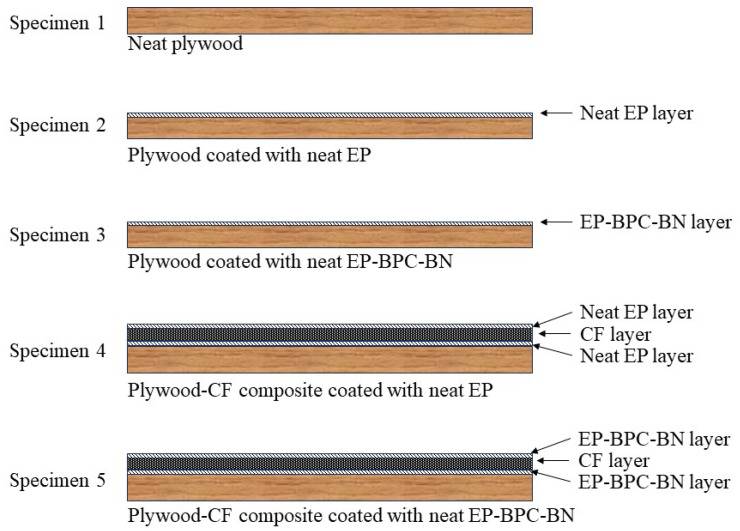
Specimens for cone calorimetry testing.

Three sets of 3 mm thick plywood specimens were prepared for the cone calorimetry testing. The first set was a neat plywood specimen, the second set of plywood specimens were coated with a layer of neat epoxy, the third set of plywood specimens were coated with EP–BPC–BN, the fourth set of plywood specimens were coated with neat EP and a layer of carbon fibre (CF) sheet was fixed to the exposed face of the plywood (CFEP), and the fifth set of plywood specimens were coated with EP–BPC–BN and a layer of carbon fibre sheet was fixed to the exposed face of the plywood (CFFREP). Both the carbon fibre–plywood composites were further coated with the corresponding EP coatings on the exposed face. Figure 12 illustrates the vertical cross-sectional view of the prepared specimens for cone calorimetry testing.

Table 4 summarises the cone calorimetry test results of neat plywood, neat EP-coated plywood, fire-retardant epoxy (FREP)-coated plywood, carbon fibre composite plywood with neat EP, and carbon fibre composite plywood with FREP. Peak heat release rate (PHRR), average heat release rate (average HRR at 180 s), and total smoke release (TSR) of the specimens were determined by cone calorimetry. Heat release rate is the rate at which fire releases energy. The average HRR was calculated from the integral of HRR over the period of heat release from the specimen. TSR is the measure of the amount of smoke produced during exposure to heat.

PHRR, average HRR, and TSR of fire retardant-coated plywood were slightly lower compared to the neat EP-coated plywood. The neat EP-coated plywood showed a 13% increase in PHRR which was reduced to a 6% increase when compared to the neat plywood. PHRR is the highest rate at which heat is released during a fire, and it indicates the intensity of the fire and how quickly a fire can spread. The result indicates the ability of the modified epoxy composite to reduce the fire growth, fire intensity, and related fire hazards of neat epoxy-coated plywood. The result also reflects the ability of the modified epoxy coating to reduce PHRR to more than half of that of the neat epoxy coating. Time to PHRR is the time in seconds to reach peak heat output that results in sustained flame spread. Time to PHRR of the FREP-coated plywood was at 60 s, 20 s earlier than the neat EP-coated plywood, and time to ignition (TTI) remained the same for both coatings.

Similarly, the TSR value of the FREP-coated plywood was lower than that of the neat EP-coated plywood. The percentage of smoke release (TSR%) from the neat EP-coated plywood was increased to 66% compared to the neat EP. However, the TSR% of the modified FREP-coated plywood was reduced to 62%. From a fire-retardant coating perspective, this data indicates a significant improvement since historically all fire-retardant coatings produce a high amount of smoke. Hence, a 4% reduction in smoke from the FREP compared to the neat EP is considered significant.

It was identified that the PHRR average HRR and TSR of fire retardant-coated CFFREP was higher than that of the neat CFEP. This is attributed to the extra volume of epoxy resin added to the CFFREP composite for proper adhesion. An equivalent volume to neat epoxy resin was insufficient to obtain complete adhesion of CF with FREP to the plywood surface. Further investigation is required to identify the compatibility and adhesion characteristics of FREP with CF on plywood surfaces.

### 3.7. Microstructure Analysis

**Figure 13:**
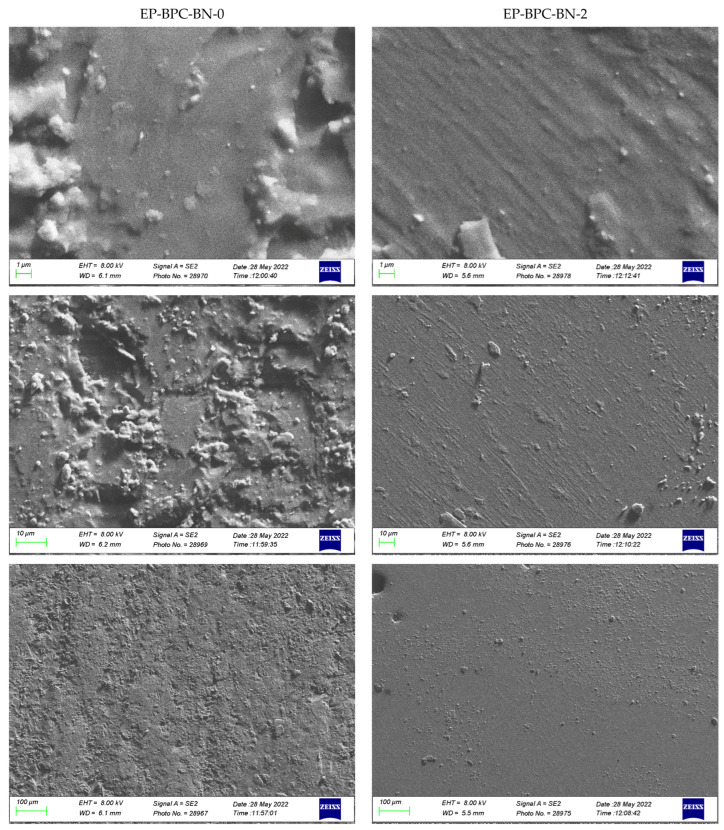
SEM images of neat EP and EP–BPC–BN composite.

The surfaces of the EP and EP-BPC-BN nanocomposites were observed with a scanning electron microscope to investigate the surface morphology and distribution state of the nanofiller in EP matrix. As shown in Figure 13 the surface of EP is relatively rough and uneven; however. the surface of EP-BPC-BN composite does not show noticeable roughness or agglomeration. It shows a good dispersion of BNNPs in the EP matrix with the help of BPC complex. However, considering the variations in magnification and grayscale of the images, and considering the low weight percentage of BNNPs in the composite matrix, these SEM characterization images are not expected to provide an accurate measure of dispersion of the nanoparticles.

## 4. Flame-Retardant Mechanism

The DSC results show that the glass transition temperature of the EP was decreased when it was mixed and cured with BPC, but when 0.2 wt% of BNNP was added to the blend, the *T_g_* of the EP–BPC blend increased to the original value of neat EP. This was attributed to the improved matrix–filler interface. The uniform dispersion of BPC-assisted BNNPs in the EP matrix exhibits a strong confinement effect on the mobility of polymeric networks.

The uniform distribution of BNNPs in the epoxy matrix acted as a thermal bridge to carry the surface heat through the epoxy matrix rapidly. The uniformly dispersed BNNPs hindered the O_2_ molecules from taking part in the combustion process [51]. The exfoliation of BNNPs in BPC complex resulted in uniform dispersion of BNNPs in the EP matrix. BPC is an ionic liquid which contains a large boron-centric anion and a proton. The composite has increased ability to conduct heat rapidly compared to the neat EP as verified by the LFA test results. Phonon transport has occurred through the H-bond between the BPC complex and the matrix via evenly dispersed BNNPs. Intermolecular hydrogen bonding is beneficial for improved phonon transport through the matrix [52,53] and hydrogen bonding can improve thermal conductivity by reducing the twisting motion of the molecular chain which improves molecular crystallinity [28,54]. Hence, uniform dispersion of BNNPs, the ionic liquid characteristics of BPC, the formation of a thermal bridge via BNNPs, and H-bonding and high thermal conductivity of the polymer composite resulted in better flame retardancy.

**Figure 14:**
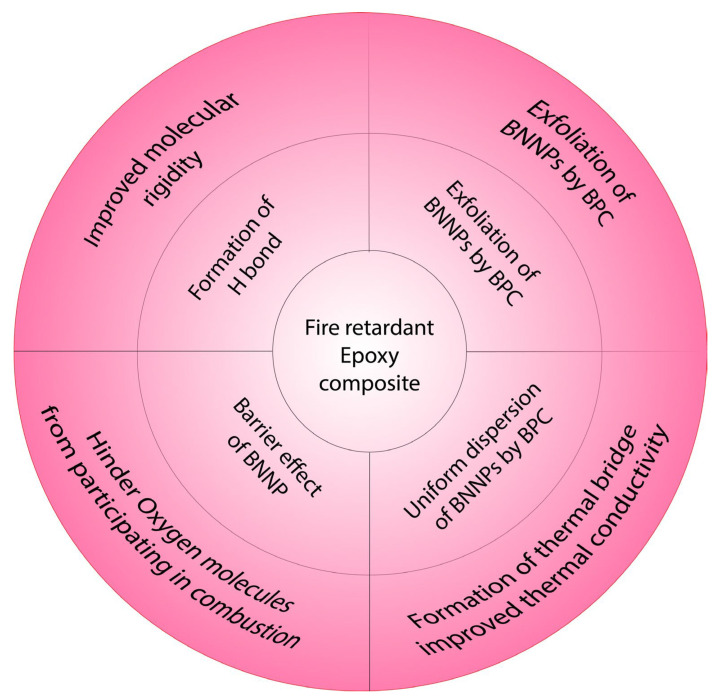
Illustration of flame-retardant mechanism.

While inspecting the post-test specimens of the UL-94 vertical burning test, it was observed that the char residue on the surface of EP-BPC-BN composite was significantly low compared to the neat EP. This was reconfirmed with Raman spectroscopy testing. The presence of a compact and efficient heat transfer network [55] has contributed to less char formation on the surface of the composite. The flame-retardant mechanism of the epoxy composite is illustrated in Figure 14.

## 5. Conclusions

It is concluded that the ionic liquid characteristics of BPC result in the formation of an ionanofluid when mixed with BNNPs. From this experiment, it is identified that the incorporation of nanomaterials—BNNPs—into the epoxy polymer matrix via the formation of ionanofluids results in enhanced material properties due to the chemical interaction between the constituent additives to the epoxy polymer chain. This finding is the novelty of this research. The remarkable fire retardancy was attributed to the synergistic effect of BNNPs and BPC complex.

The resultant composite demonstrated that the modification of BN with the polyol complex imparted a low smoke and char formation to the modified epoxy composites. SEM images indicated uniform dispersion and reduced agglomeration of BNNPs in the EP matrix. The FTIR spectrum of the burned composite highlighted the hydroxyl functionalised BNNPs in the EP matrix, which showed proof of hydrogen bonding. Laser flash analysis results indicated improved thermal diffusivity of the BNNP composites and DSC results confirmed the improved glass transition temperature. The product may find its place in thermal management applications.

## Figures and Tables

**Table 1:** EP–BPC–BN mixing ratios.

Sample ID	DGEBA Resin (g)	25% BPC (g)	DDM (g)	BNNP (g)	BN (wt%)	BPC (wt%)	BPC–BN (wt%)
EP-BPC-BN-0	10	0	3	0	0	0	0
EP-BPC-BN-1	10	2	3	0	0.00	13.33	13.33
EP-BPC-BN-2	10	2	3	0.03	0.20	13.33	13.51
EP-BPC-BN-3	10	2	3	0.06	0.41	13.33	13.68
EP-BPC-BN-4	10	2	3	0.09	0.60	13.33	13.86
EP-BPC-BN-5	10	2	3	0.12	0.80	13.33	14.03

**Table 2:** UL94 test results of neat EP and EP–BPC–BN composites.

Sample	UL-94
EP-BPC-BN-0	No rating (NR)
EP-BPC-BN-1	V-1
EP-BPC-BN-2	V-0
EP-BPC-BN-3	V-0

**Table 3:** Comparison of FTIR spectra of burned EP, EP–BPC complex, and EP-BPC-BN composites.

Wave Number (cm^−1^)	Intensity			Caused by	Effect	
	EP-BPC-BN-0	EP-BPC-BN-1	EP-BPC-BN-2			
1370	None	None	Low	B-N tensile vibration	Non-combustible BNNPs in the composite	
811	None	None	Low	B-N-B bending vibrations	Non-combustible BNNPs in the composite	
1290	None	None	Low	B-O deformation vibration	Functionalisation of BNNPs with OH	
3100–3590	Low	Medium	High	O-H tensile vibrations	Functionalisation of BNNPs with OH	
658	None	Medium	Medium	Boron spiro structure vibration	Presence of BPC complex in the composite	
2931	Low	High	Medium	C-H stretching vibrations	Presence of BPC complex in the composite	
2850	Low	High	Medium	C-H stretching vibrations	Presence of BPC complex in the composite	

**Table 4:** Cone calorimetry test results of the EP- and FREP-coated plywood and CF composite plywood.

Composites	Time to PHRR (s)	PHRR (Kw/m^2^)	PHRR% Increase (%)	Average HRR (Kw/m^2^)	TSR (m^2^/m^2^)	TSR % Increase (%)	TTI (s)
Neat plywood	55	317	-	112	68	-	22
Neat EP-coated plywood	80	358	13	111	113	66	23
FREP-coated plywood	60	335	6	109	110	62	23
Carbon fibre-reinforced plywood composite							
Neat CFEP	80	236	-	98	145	-	26
CFFREP	90	285	21	113	207	43	23

## Data Availability

The original contributions presented in this study are included in the article. Further inquiries can be directed to the corresponding author.
